# Pseudohomogeneous metallic catalyst based on tungstate-decorated amphiphilic carbon quantum dots for selective oxidative scission of alkenes to aldehyde

**DOI:** 10.1038/s41598-021-83863-0

**Published:** 2021-02-24

**Authors:** Aram Rezaei, Leila Hadian-Dehkordi, Hadi Samadian, Mehdi Jaymand, Homa Targhan, Ali Ramazani, Hadi Adibi, Xiaolei Deng, Lingxia Zheng, Huajun Zheng

**Affiliations:** 1 Nano Drug Delivery Research Center, Health Technology Institute, Kermanshah University of Medical Sciences, Kermanshah, Iran; 2 Department of Organic Chemistry, Faculty of Chemistry, University of Razi, 67149-67346 Kermanshah, Iran; 3 Department of Chemistry, University of Zanjan, Zanjan, Iran; 4 Pharmaceutical Sciences Research Center, Health Institute, Kermanshah University of Medical Sciences, Kermanshah, Iran; 5 Department of Applied Chemistry, Zhejiang University of Technology, Hangzhou, 310032 China; 6 State Key Laboratory Breeding Base of Green Chemistry Synthesis Technology, Zhejiang University of Technology, Hangzhou, 310032 China

**Keywords:** Catalyst synthesis, Heterogeneous catalysis, Homogeneous catalysis

## Abstract

Herein, we present an interesting role of tungstate-decorated amphiphilic carbon quantum dots (A-CQDs/W) in the selective oxidative cleavage of alkenes to aldehydes. In this work, for the first time, we disclose an unprecedented tungstate-based oxidative system incorporating A-CQDs as a bridge to the homogeneous catalyst for selective and efficient cleavage of a wide substrate scope of alkenes into aldehydes. The A-CQDs/W were synthesized via a one-step hydrothermal synthesis approach using 1-aminopropyl-3-methyl-imidazolium chloride and stearic acid for the surface modification, following by anion-exchange to immobilize WO_4_^–2^ to A-CQDs. The A-CQDs/W act as a pseudohomogeneous metallic catalyst (PMC) for selective oxidative scission of alkenes under phase transfer catalysts (PTC) free condition without over oxidation to acids, using water and H_2_O_2_ as a green oxidant. Thanks to the sub-nanometric size and novel engineered chemical structure, this PMC and reactants are in the same phase, besides they can be easily isolated from each other by extraction processes. The synthesized PMC exhibited excellent solubility and stability in various solvents. Interestingly, the system’s high conversion efficiency was preserved even after eight catalytic cycles indicating the recyclability of the synthesized PMC. We believe that this study provides a significant and conceptually novel advance in oxidative cleavage chemistry.

## Introduction

The presence of sub-nanometric carbonaceous supports relied on carbon quantum dots (CQDs) in the structure of a metal-based catalyst may add the advantage of both heterogeneous and homogeneous catalysis to it, providing a new generation of recoverable and highly active metal-based catalysts called pseudohomogeneous metallic catalyst (PMC)^1^.

Metal-based catalyst is a heterogeneous catalytic system that has a significant role in transforming organic compounds into high-value chemicals. Also, support has a crucial role in heterogeneous catalysis, making a major contribution to mechanical stability, recyclability, and chemical stabilization. In other words, the most significant cons of these heterogeneous catalysis are limited selectivity and activity because of the lack of being in the same phase as the reactants^2^.

The CQD is a promising new material to serve as a catalyst or support due to its sub-nanometric size and tunable phase behavior, which operates as a bridge to recoverable homogeneous system to solve conventional support problem^3^. Moreover, CQDs with tunable functionalities, stable suspension, and unique physicochemical properties, for instance, chemical and thermal resistance, high intrinsic carrier, active surface area, ease of synthesis and environmental and economic friendliness, promise talent support for a wide range of metal-based catalysis as an alternative to conventional heterogeneous supports^4–6^.

Compared with other transition metals, tungsten presents unique physicochemical properties such as availability, low-cost, non-toxicity, oxygen-carrying ability, and enough activity toward H_2_O_2_^7^. Therefore, a great number of oxidizing W-H_2_O_2_ systems were developed to perform green oxidation reactions. So, numerous valuable oxidation processes such as alcohol, sulfide and amine oxidation and alkene epoxidation rely on tungsten-based catalysts heavily^8^.

**Scheme 1:**
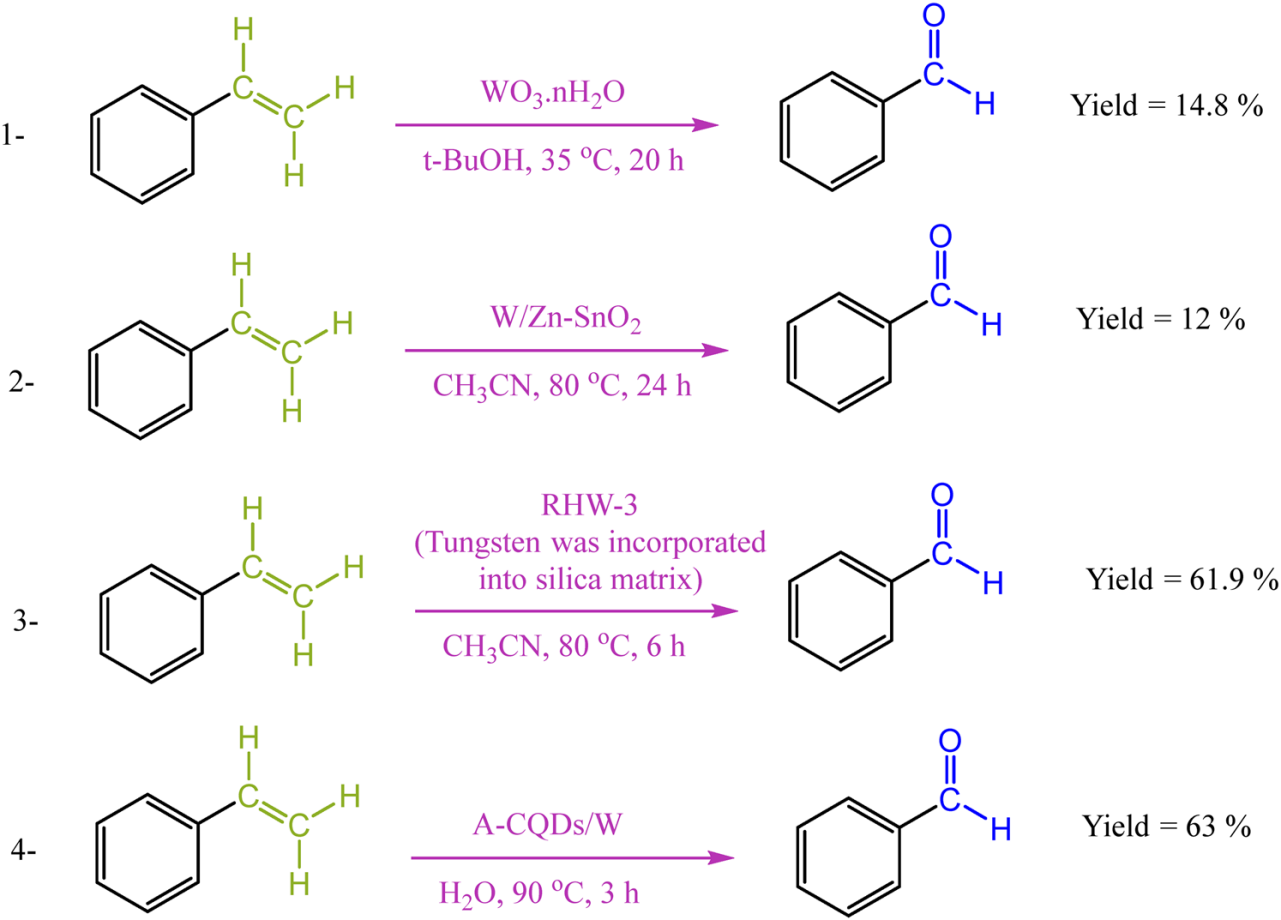
Comparison of the activity of A-CQDs/W catalyst in oxidative cleavage of styrene to benzaldehyde with the reported tungsten-based catalysts.

What might be surprising is that there are only a very few reports on a significantly important tungsten-based oxidative cleavage reaction of alkenes to aldehydes. These rare examples are also suffering from limited substrate scope, multi-step catalyst synthesis, low conversion, selectivity, and limited reuse times (Scheme 1)^9–11^. For example, although tungsten supported on MCM-41 or TiO_2_ allows the oxidative cleavage in the presence of H_2_O_2_ as the oxidant to produce aldehydes selectively, these studies were limited to cyclopentene as the only substrate^12,13^. Other tungsten catalytic systems with SBA-15-type material^14^, modified organic–inorganic hybrid silica^15^, and H_3_PW_4_O_24_/PEHA/ZrSBA-15 system^16^, as supports were also reported. However, these systems not only lead to over oxidation to carboxylic acids, but also presence of progressive contamination, makes these catalytically processes uneconomical.

In 2019, we reported the ionic liquid (IL) modified CQDs as an adsorbent and stabilizing support for WO_4_^2−^ ions (CQDs@IL/WO_4_^2−^). In the presence of CQDs@IL/WO_4_^2−^, oxidation of alcohols with 30% H_2_O_2_, as the oxidant, could be conducted in CH_3_CN/H_2_O solvent mixture at 90 °C to afford the aldehydes and ketones in high to excellent yields and selectivities^4^. Unfortunately, in the biphasic conditions, the hydrophilic nature of these amazing materials is unfavorable for the reaction of the lipophilic substrates. In subsequent work, we successfully omitted organic solvents in the reaction media by adding a hydrophobic long-chain part, dodecyl amine, to the hydrophilic CQDs@IL/WO_4_^2−^ complex. This innovative amphiphilic catalyst was profitably applied in the oxidation of alcohols in the presence of 30% H_2_O_2_ in mild conditions providing aldehydes in high yields and 100% selectivity. Therefore, unlike the first system for alcohol oxidation reaction, the second one is conducted in water without additional organic solvents^3^. Our previous studies on the IL-modified CQDs as exceptional catalyst support for biphasic conditions have prompted us to develop this catalyst class in another important organic transformation.

Recently, our group introduced a novel oxidative scission system based on A-CQD as a pseudohomogenous carbocatalyst. This metal-free catalyst in the presence of H_2_O_2_ selectively converted alkenes to aldehyde with good catalytic results^17^. Afterwards, we have made a hard attempt to find an efficient strategy to increase conversion and yield in selective oxidative cracking of alkenes to aldehydes.

Herein, A-CQDs introduce as superior support to immobilize WO_4_^2−^ ions through an anion exchange reaction to form A-CQDs/W. To the extent of our knowledge, for the first time the A-CQDs/W provide new horizons for selective cracking of alkene families to aldehydes compounds in the presence of H_2_O_2_ as a green oxidant without additives or PTC in water. Admittedly, this newly presented catalyst benefit from the various advantages, such as wonderfully low tungsten leaching and providing a pseudohomogeneous catalysis system, which resulted in ease of accessibility for reaction substrates. In this work, we demonstrated that through a combination of tungsten ions and A-CQDs, a significantly novel and active PMC was designed for the selective oxidative cleavage of a broad scope of olefins to aldehydes.

## Experimental

### Materials and Apparatus

All chemicals and solvents were supplied from Fluka (Switzerland) or Merck (Germany). We used an ESCALab MKII (Thermo Fisher Scientific, USA) spectrometer with Al Kα (1.4866 keV) as the X-ray source to determine X-ray photoelectron spectroscopy (XPS). The ^1^H NMR and ^13^CNMR analyses were carried out with a BRUKER DRX-250 AVANCE spectrometer at 250.0 MHz. The optical emission spectrometer inductively coupled plasma (Varian Vista MPX ICP-OES Axial) was used to measure content of W in the catalysts. X-ray diffraction (XRD) spectra were obtained on a Siefert XRD 3003 PTS diffractometer with Cu Kα radiation (λ = 1.54 Å). Thermogravimetric analysis (TGA) was recorded using a TGA Q 50 analyzer under N_2_ flow at a heating rate of 10 °C min^−1^. The optical characteristics of samples were measured by Shimadzu UV 2100 151PC UV–Visible spectrophotometer at room temperature. Field emission scanning electron microscope (FE-SEM) imaging and energy-dispersive X-ray spectroscopy (EDX) analysis were carried out on a SIGMA VP 500 (Zeiss) microscope equipped with an EDX measurement system. A Philips EM10C 200 kV microscope was used to record transmission electron microscopy (TEM) images. The fourier-transform infrared spectroscopy (FT-IR) spectra of the samples were recorded with the KBr pellet method by PerkinElmer PE-1600-FTIR spectrometer.

### Synthesis procedure of A-CQDs functionalized with the 1-aminopropyl-3-methyl-imidazolium chloride ([APMim][Cl]) and stearic acid (SA)

Firstly, the [APMim][Cl] was synthesized based on our previous procedure^3,4^. Subsequently, a mixture of citric acid (3 mmol), as a sole carbon source, SA (1 mmol) and [APMim][Cl] (4.5 mmol), as the surface modifiers were dissolved in 15 mL of H_2_O:EtOH (1:2 v/v), then ammonia added to the prepared solution drop by drop to adjust pH to 8–9. The obtained solution was sonicated for 5 min and the milky solution was hydrothermally treated in an autoclave under N_2_ at 200 °C for 4 h. Following the hydrothermal treatment, the autoclave was cooled to room temperature, and then purified by 100 Dalton dialysis membrane.

### Synthesis of A-CQDs/W catalyst

The obtained A-CQDs dispersion was sonicated for 5 min and then, 2 mL H_2_O containing Na_2_WO_4_·2H_2_O (2 mmol) added dropwise and stirred at RT overnight. In this step, the color of the reaction mixture changed from initially reddish-brown to light brown. The resulting mixture was centrifuged at 16,000 rpm for 10 min to eliminate any large dots and agglomerates, and then a dialysis process through a dialysis membrane (100 Da) was conducted for 48 h to remove the remaining molecular precursors. Ultimately, a brown powder was obtained after lyophilization of dialysates.

### General procedure for selective oxidative cleavage of C=C bonds of alkenes catalyzed by A-CQDs/W

The catalysis experiments for the A-CQDs/W were carried out in H_2_O, using a 1:3 substrate:H_2_O_2_ mole ratio. The A-CQDs/W (1 mol % of W) was poured into a round‐bottom flask equipped with a reflux condenser, following the addition of alkene substrate (1 mmol). Then, H_2_O_2_ (35% aqueous solution) was gradually added over about 1 h under stirrer at 90 °C. The mixture was stirred for a further 3 h at 90 °C. After detecting the completion of the reaction by TLC, the mixture was allowed to cool to RT. Then, the oxidative cleavage product was extracted with water/ethyl acetate. In a way that, first the reaction mixture was diluted with water/ethyl acetate and then transferred into a separatory funnel. After three times extraction with ethyl acetate, the upper and bottom layer were separated. Then, the extracted catalyst in the bottom layer was dried overnight to reuse in another run under optimal conditions. On the other hand, the organic phase containing unreacted reactants and the products were evaporated and purified by employing a silica gel column chromatography (EtOAc/petroleum ether) to obtain the desired aldehydes. Finally, relied on the isolated products, the yields are reported.

### Quantum yield measurement

The photoluminescent quantum yields (QY) of the A-CQDs were determined in reference to quinine sulfate. QY were calculated according to the following equation:

$$Q_{x} = Q_{{st}} \left( {\frac{{M_{x} }}{{M_{{st}} }}} \right)\left( {\frac{{\eta _{x} }}{{\eta _{{st}} }}} \right)^{2}$$

where Q is the quantum yield, ƞ is the refractive index of the solution and M is the gradient from the linear regression. The subscripts ‘st’ and ‘x’ refer to standard fluorescent agent and sample, respectively.

## Results and discussion

### Synthesis and characterization of the catalyst

**Scheme 2:**
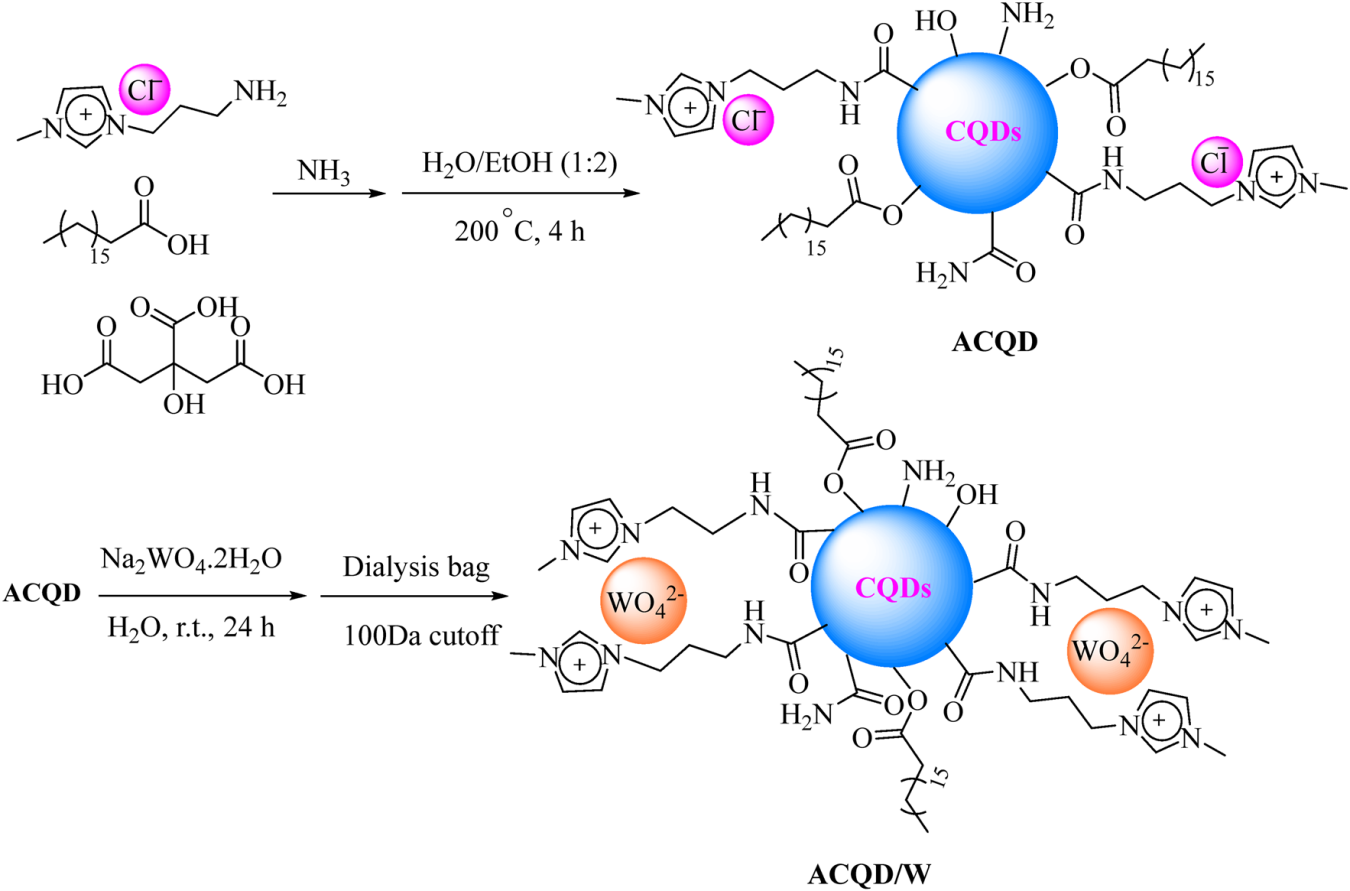
Schematic procedure for the synthesis of the A-CQDs/W catalyst.

The synthesis route of A-CQDs/W is shown in Scheme 2 and the details are described in the experimental section. At the outset, [APMim][Cl] was prepared based on the procedure investigated in our previous literature^4^. To prepare A-CQDs, starting materials underwent hydrothermal treatment at 200 °C for 4 h. Afterward, the WO_4_^2−^ ions were immobilized on the surface of modified CQDs by a facile anion-exchange reaction of A-CQDs with Na_2_WO_4_.2H_2_O (Scheme 2).

FT-IR spectroscopy analysis was used to elucidate the different functional groups present on the surface of A-CQDs and A-CQDs/W samples (Fig. S1). The FT-IR spectrum of the A-CQDs exhibits several characteristic absorption bands of oxygen-containing functional groups. The broad and intense absorption bands around 3230–3560 and 3024–3390 cm^−1^ are assigned to N–H stretching and O–H stretching vibrations, respectively. It is noteworthy that the amide carbonyl stretching mode appears at 1680 cm^−1^, C–O–C peak at 1215 cm^−1^, the esteric C-O stretching at 1172 cm^−1^, and C–O stretching vibrations at 1050 cm^−1^^18^. It can also be observed that there are bands in the range of about 1180 and 1384 cm^−1^, which are due to the C–N stretching vibration and methylene group deformation vibrations from SA and [APMim][Cl]^19,20^. The bands at 2854, 2920 and 1452 cm^−1^ also reveal the symmetric, asymmetric stretching, and scissoring vibration modes from –CH_2_ groups of these two moieties^21^. The band at 621 and 1620 cm^−1^ corresponds to C–H bending in the imidazolium ring and unoxidized sp^2^ C=C stretching, respectively^22^. Therefore, the FT-IR results showed the functionalization of A-CQDs with both hydrophilic functional groups such as ester, the imidazolium ring, amide, hydroxyl, and acidic groups, as well as hydrophobic alkyl groups in SA as modifier agents. After anion-exchange of A-CQDs with Na_2_WO_4_, the FT-IR spectrum of the A-CQDs/W (Fig. S1) also exhibits a new characteristic band around 860 cm^-1^ which is assigned to the W=O stretching frequency in WO_4_^2−^^23,24^.

The organic functional groups on the surface of A-CQDs/W were further analyzed by TGA in the temperature range of 30–700 °C under nitrogen as an inert atmosphere at a rate of 10 °C min^−1^. Figure S2 represents the information on the degradation patterns of A-CQDs and A-CQDs/W. TGA results indicate that the A-CQDs and A-CQDs/W samples showed a weight loss of 70.1% and 52.4%, respectively, below 700 °C^25^. The A-CQDs/W was found to be stable up to 165 °C with a weight loss of only ∼ 6% which might be related to the evaporation of the remaining solvent. As the temperature increases from 165 to 276 °C a weight loss of 11.34% is observed, which can be due to the decomposition of carboxyl and hydroxyl functional groups^26^. Moreover, a weight loss was observed in the temperature range from 276 to 472 °C (22.66%) due to the removal of the SA and the [APMIm][Cl] attached to the surface of A-CQDs. While in the case of the A-CQDs, an approximate weight loss of ∼ 4% can be seen up to 120 °C. As the temperature increases, a dramatic weight loss of ∼ 55% between 120 to ∼ 460 °C was observed. This weight loss can be due to steam release and decomposition of oxygen-containing functional groups and degradation of the covalently grafted organic addends, including SA and the [APMIm][Cl]^27,28^. For both samples, the later weights loss above the temperature of 480 °C can be attributed to the decomposition of the graphene-like carbon network, which is in a good agreement with the behavior of other carbon-based materials^29,30^.

**Figure 1:**
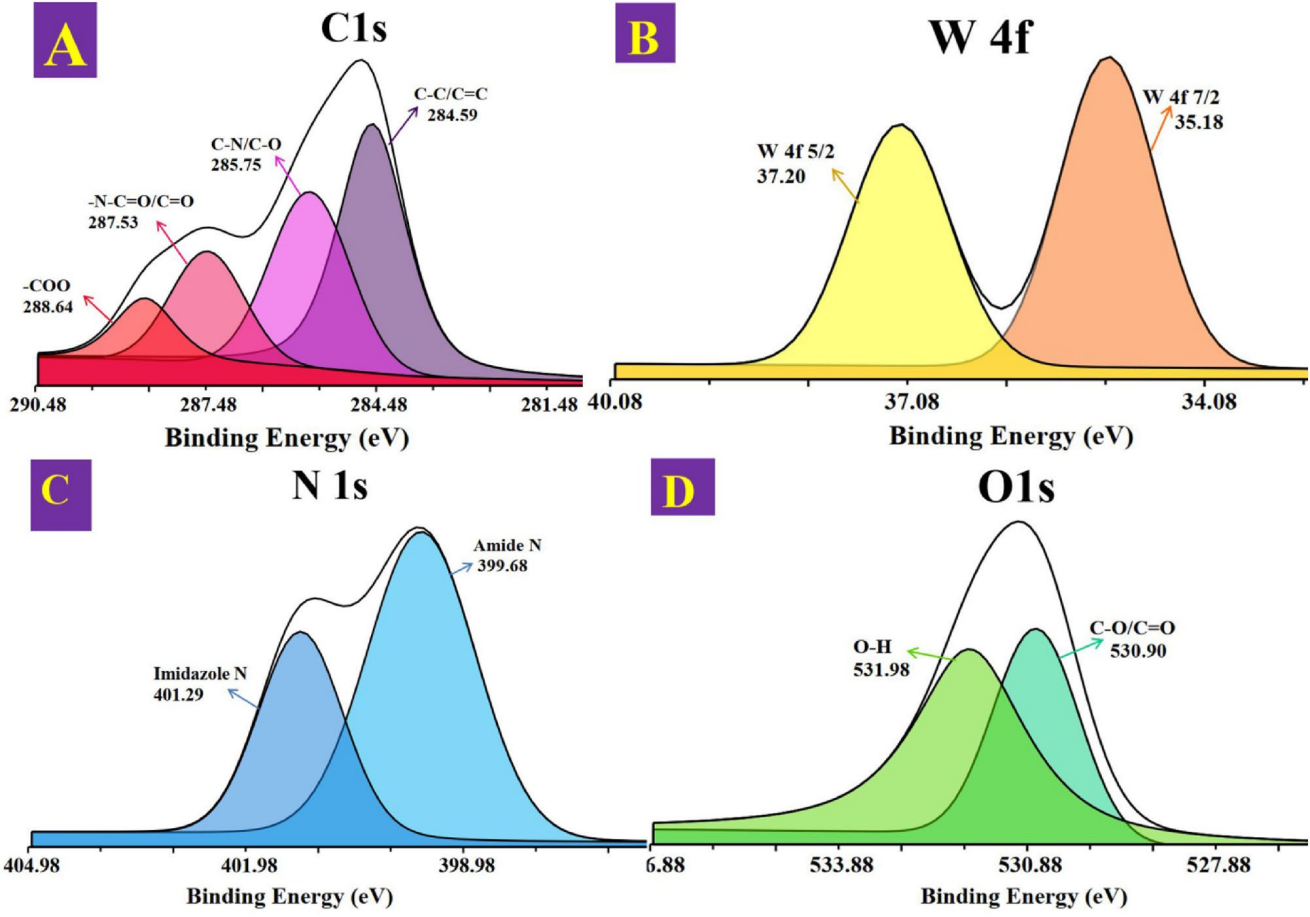
High-resolution XPS spectra of the A-CQDs/W sample: **(A)** C 1s XPS spectra (**B**) W 4f XPS spectra, **(C)** N 1s XPS spectra and **(D)** O 1s XPS spectra.

Further evidence for the formation of the A-CQDs/W was obtained through XPS results, which is an excellent analysis for understanding the surface chemical composition of catalyst and the oxidation state of the tungsten on it. The XPS results of the A-CQDs/W reveal the presence of C, O, N and W elements with atomic percentages of 70.68, 17.47, 10.64 and 1.20, respectively, indicating the successful formation of the A-CQDs/W. The high-resolution C 1s spectrum contained four peaks at 284.59, 285.75, 287.53, and 288.64 eV, which corresponded to C–C/C=C , C–N/C–O, –N–C=O/C=O, and –COO groups, respectively (Fig. 1A)^31^. Besides, the high-resolution XPS spectrum for W 4f is divided into W 4f_7/2_ and W 4f_5/2_ peaks (Fig. 1B), where the peaks at 35.18 and 37.20 eV attributed to the reported binding energy for W^6+^^32–34^. Moreover, the high-resolution N 1s spectra is depicted in Fig. 1C and two dominant peaks located at 399.68 and 401.29 eV related to amide and imidazole ring, respectively^35^. The high-resolution O 1s spectrum also displays two typical peaks around 530.90 and 531.98 eV, which are resulted from the oxygen bonds of C–O/C=O and O–H species, as shown in Fig. 1D^3,4,36^. Not only do these results further prove that the A-CQDs are successfully formed and SA and [APMIm][Cl] are covalently grafted to the surface of CQDs, but also indicate that WO_4_^2−^ ions are immobilized on the surface of A-CQDs, which correspond to the IR results.

^1^H and ^13^C NMR analysis are applied to characterize the structure of A-CQDs and the results are presented in Figs. S3A,B, respectively. Figure S3A shows a characteristic ^1^H NMR spectrum of the A-CQDs, a set of peaks at 1.18–2.46 ppm corresponds to the CH_2_ and CH_3_ groups in the SA and [APMIm][Cl] parts^3^. While, the 3.13–4.06 ppm peaks were ascribed to the protons adjacent to electron-withdrawing groups (the C=O and N) in the SA and [APMIm][Cl] parts. Furthermore, the peaks in the range of 7.32–8.66 ppm can be well assigned according to the sp^2^ protons of the imidazolium ring. In ^13^C NMR spectrum (Fig. S3B), the peaks in the range of 20–60 ppm can be ascribed to sp^3^ carbons of the alkyl chain from SA and [APMIm][Cl], and the peaks at 60–80 ppm were assigned to the sp^3^ C atoms attached to electron-withdrawing groups. Furthermore, the imidazole ring carbons were inhabited between 122 and 136 ppm. In addition, the peaks located at 177–181 ppm are related to the carbonyl carbons. Overall, the NMR spectroscopic data are in general agreement with IR results and support the expected structure of A-CQDs^3,36^.

**Figure 2:**
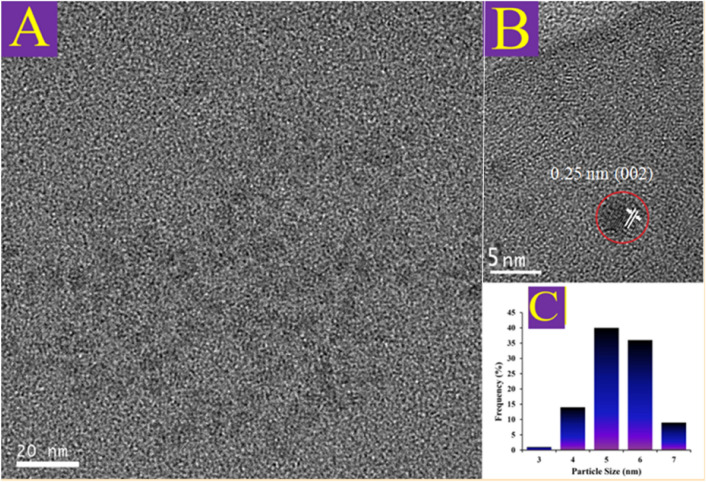
**(A)** TEM image of the A-CQDs/W; **(B)** The 5 nm resolution TEM image of A-CQDs/W, and **(C)** The size distributions of the A-CQDs/W.

Figure S4 displays FE-SEM images of the as-prepared sample with 100 and 200 kx magnifications. It can be seen that the particles of A-CQDs/W are spherical shapes with diameters below 10 nm. The EDX technique was employed to elucidate the distribution and constituent elements of the A-CQDs/W. The EDX result provides the elemental composition for the A-CQDs/W (C, O, N, and W) and elemental mapping shows that the elements were uniformly distributed over the nanocatalyst (Fig. S5)^3,4,17^. TEM (HR-TEM) analysis was utilized for a detailed study of the surface morphology, size, and shape of the A-CQDs/W catalyst, and the results are depicted in Fig. 2A–C. The HR-TEM image confirms the spherical shape of the A-CQDs/W, which is in agreement with the FE-SEM results. As a result, the hydrothermally prepared A-CQDs/W NPs are uniform and well-dispersed with an average size in the range of 4–6 nm^3,17^.

Figure S6A, B depicts the fluorescence response of the A-CQDs/W sample with increasing excitation wavelengths in the range of 300 to 390 nm. The sample displayed the maximum emission at around 432 nm under excitation with 340 nm. In addition, the A-CQDs/W showed 87% of photoluminescent quantum yield (QY). We also used fluorescence spectroscopy to determine the stability of A-CQDs/W vs time by measuring the amount of fluorescence intensity changes of CQDs over 3 months. The results show that the rate of fluorescence reduction during this period was about 6% (Fig. S6C). The UV–vis absorption spectra of A-CQDs and A-CQDs/W samples are also presented in Fig. S6D. The A-CQDs and A-CQDs/W samples exhibited two peaks at 238 nm and 368 nm, which could be attributed to the π–π* transition of an aromatic π-system and n–π* transition of carbonyl groups, respectively^37,38^.

The XRD analysis of the A-CQDs/W is shown in Fig. S7. The single broad peak that appeared at 2θ ~ 24° is attributed to the (002) plane of the amorphous graphite-like structure of the small size samples derived from the citric acid’s carbonization^3^. The in-plane diffraction correlated with (101) plane of the graphene-like structure of C-dot led to the peak located at about 47°. Moreover, the increased full width half maximum (FWHM) of the pattern and its decreased intensity show the amorphous nature of these A-CQDs/W as a result of nitrogen and oxygen functional groups on its surface^39^.

### The investigation of the catalytic activity of A-CQDs/W in oxidative cleavage reaction of alkenes

Aldehydes form an inescapable part of numerous industrial processes because of their essential applications. In biology/medicine concept, they are applied as the precursor to the synthesis of disinfectants, tissue fixating agents, flavors, perfumes, germicides, insecticides, bee repellent, fungicides, preserving biological specimens, and beauty products. In the chemical industry, they are typical building blocks to produce other necessary chemicals used in dyes, tanning, polymeric products, organic acids, adhesives, coatings, resins, and pyridine derivatives. They are also used in photography and drug testing^40,41^. As a result, aldehydes are crucial in the synthesis of various organic compounds and industry^42–46^. Therefore, a study about the synthesis of aldehydes forms a critical part of organic chemistry. Unfortunately, despite immense efforts have been made in this scope, the current oxidative cleavage methods are usually suffering from many disadvantages, such as the requirement to use toxic organic solvents, harsh conditions, limited substrates, need to PTC, expensive catalyst, and excessive amounts of oxidants along with low selectivity, producing excessive byproducts, and over oxidation to acids^39^.

To overcome these obstacles, we proposed A-CQDs/W as the alternative over the conventional catalysts. The catalytic activity of A-CQDs/W was firstly evaluated in oxidative cleavage of coumarin-3-carboxylic acid as the starting substrate, owing to their excellent pharmaceutical value and universal interest. In order to determine the optimal experimental conditions, various parameters, including the catalyst amount, presence of PTC, solvent system, the H_2_O_2_/substrate molar ratios, temperature and additive effect were investigated.

At the outset, the effect of A-CQDs/W on the decomposition of hydrogen peroxide was studied. Interestingly, during this process, no oxygen gas is released. As seen in Table 1, in the presence of H_2_O_2_ only (entry 1), and also H_2_O_2_ and Na_2_WO_4_, without any A-CQDs support (entry 2), virtually no aldehyde was produced. Then the reaction was examined in the absence of hydrogen peroxide and in the presence of A-CQDs/W (1 mol %) at 90 °C to examine the role of H_2_O_2_ in this catalytic system. Appropriately, no conversion of coumarin-3-carboxylic acid was achieved (entry 3), so it is clear that H_2_O_2_ has a crucial role in this oxidation reaction. A-CQDs without tungsten were also active in the oxidative cleavage reaction (entry 4 and 5)^17^. In the following, the coumarin-3-carboxylic acid was then treated with 0.25, 0.5, 1 and 1.5 mol % of W in the A-CQD/W catalyst in the presence of H_2_O_2_ (3 mmol) (entries 6–9). The best result in terms of conversion and yield (95% and 86%, respectively) was obtained by using 1 mol % of W in the A-CQDs/W (entry 8). In the subsequent step, we assessed the effect of cetyltrimethyl ammonium hydrogen sulfate as a PTC on the catalytic performance, and we found no significant differences in the conversion of the substrate and the yield of products in the presence or absence of PTC (entry 10). It could be because of this fact that the A-CQDs simultaneously consists of hydrophilic and hydrophobic moieties; thus, the presence of PTC in the catalytic process is eliminated. The amphiphilicity of catalyst was further confirmed by dispersity of A-CQDs/W in various solvents such as water, EtOH, DMF, acetonitrile, toluene. The high blue emission of the A-CQDs/W dispersed in each of the above solvents under 365 nm UV irradiation shown in Fig. S8^3^.

**Table 1:** The effects of various reaction conditions on the oxidative cleavage of coumarin-3-carboxylic acid with A-CQDs/W.


Entry	Catalyst	PTC (mmol)	Solvent	H_2_O_2_/Substrate (mmol/mmol)	T (°C)	Conversion (%)^a^	Yield (%)^b^
1	–	–	H_2_O	3/1	90	Trace	Trace
2	Na_2_WO_4_ (0.02 mmol)	–	H_2_O	3/1	90	Trace	Trace
3	A-CQDs/W (1 mol%)	–	H_2_O	0	90	Trace	Trace
4	A-CQDs (1 mg)	–	H_2_O	3/1	90	73	61
5	A-CQDs (10 mg)		H_2_O	3/1	90	75	63
6	A-CQDs/W (0.25 mol%)	–	H_2_O	3/1	90	79	69
7	A-CQDs/W (0.5 mol%)	–	H_2_O	3/1	90	88	75
8	A-CQDs/W (1 mol%)	–	H_2_O	3/1	90	95	86
9	A-CQDs/W (1.5 mol%)	–	H_2_O	3/1	90	86	73
10	A-CQDs/W (1 mol%)	0.1	H_2_O	3/1	90	87	73
11	A-CQDs/W (1 mol%)	–	H_2_O	2/1	90	79	68
12	A-CQDs/W (1 mol%)	–	H_2_O	4/1	90	71	62
13	A-CQDs/W (1 mol%)	–	H_2_O/EtOH	3/1	90	90	83
14	A-CQDs/W (1 mol%)	–	H_2_O/Toluene	3/1	90	88	57
15	A-CQDs/W (1 mol%)	–	H_2_O/CH_3_CN	3/1	90	71	20
16	A-CQDs/W (1 mol%)	–	Pure EtOH	3/1	90	80	61
17	A-CQDs/W (1 mol%)	–	H_2_O	3/1	RT	trace	trace
18	A-CQDs/W (1 mol%)	–	H_2_O	3/1	50	21	17
19	A-CQDs/W (1 mol%)	–	H_2_O	3/1	70	84	67

Reaction conditions: Coumarin-3-carboxylic acid (1 mmol), H_2_O (1.5 mL), 90 °C, 3 h.

^a^Conversions were calculated based on initial mmol of coumarin-3-carboxylic acid.

^b^Isolated Yields.

In order to obtain the best H_2_O_2_/substrate molar ratio, different H_2_O_2_/coumarin-3-carboxylic acid molar ratios (2:1, 3:1 and 4:1) were considered in the presence of 1 mol% A-CQDs/W (entries 11, 8 and 12). Increasing the H_2_O_2_/coumarin-3-carboxylic acid ratio from 2:1 to 3:1, raises the conversion from 79 to 95%, and the yield from 68 to 86% (entries 11, 8), but the further increment of H_2_O_2_ amount resulted in reducing both conversion and yield (entry 12). Therefore, it is clear that the 3:1 molar ratio of H_2_O_2_/substrate is the best one to obtain the maximum conversion and yield (entry 8). To choose an optimum solvent, we started with the H_2_O (entry 8). Then the reactions were conducted in H_2_O/EtOH, H_2_O/toluene and H_2_O/CH_3_CN solvent mixtures and also in pure EtOH, but no better results were obtained (entries 13–16, respectively). Then we attempted to improve the substrate's conversion and increase the yield by adjusting the temperature (entries 17–19). The best results with respect to both conversion and yield were obtained using 1 mol % of A-CQDs/W and 3 equiv of H_2_O_2_ in 1.5 mL of H_2_O at 90 °C during 3 h reaction time (entry 8).

Table 2 we presents the results of *trans*-stilbene oxidation with A-CQDs/W in the presence of aqueous hydrogen peroxide in various conditions. The reaction is best carried out at 90 °C (entry 1). But, these results show that 70 °C temperature cannot be used when a high conversion of aldehyde is desired (entry 2). In addition, when H_2_O_2_/substrate molar ratio is 2:1, 43% benzaldehyde was achieved (entry 3). Besides, the conversion of benzaldehyde is not satisfactory in A-CQDs/W (0.5 mol%) (entry 4).

**Table 2:** Investigation of oxidative cleavage of *trans*-stilbene under optimum conditions in the presence of acidic additives.


Entry	Catalyst	H_2_O_2_/Substrate (mmol/mmol)	T (°C)	Con. (%)^a^	Yield (%)^b^
1	A-CQDs/W (1 mol%)	3/1	90	63	58
2	A-CQDs/W (1 mol%)	3/1	70	50	45
3	A-CQDs/W (1 mol%)	2/1	90	49	43
4	A-CQDs/W (0.5 mol%)	3/1	90	52	43

Reaction conditions: *trans*-Stilbene (1 mmol), H_2_O (1.5 mL), A-CQDs/W (1 mol %), 3 h.

^a^Conversions were calculated based on initial mmol of *trans*-stilbene.

^b^Isolated Yields.

The substrate scope in the selective oxidative cleavage of C=C bonds of a series of structurally diverse substrates was also examined (Table 3). In the first place, 3-substituted coumarins were examined in the oxidative cleavage of C=C bonds (entries 1–3). Wonderfully, the 3-substituted coumarin derivatives in the presence of A-CQDs/W and aqueous H_2_O_2_ gave the oxidative cleavage products in two steps. First, the scission of electron-deficient C=C bond of coumarin, followed by hydrolyzing the intermediate to produce the corresponding salicylaldehyde derivatives in good to high yields without over oxidation to acid. It is worth noticing that the oxidative cleavage reaction of coumarins is along with the catalyst for cleaving C–O bond. In this line, after obtaining the aforementioned high results for coumarin-3-carboxylic acid (1a), oxidation on 8-methoxycoumarin-3-carboxylic acid (1b) was also tested and gave its corresponding cleavage product, *ortho*-vanillin (2b), in 69% yield (entry 2). Delightfully, 3-acetylcoumarin (1c) as a nonacidic coumarin substrate could also be oxidized into the corresponding aldehyde (2a) with a relatively high yield (63%, entry 3).

**Table 3:** Oxidative cleavage of alkenes to corresponding aldehydes in the presence of A-CQDs/W catalyst.

Entry	Structure		Product	Conversion (%)^a^	Yield (%) ^b^
1				95	86
2				78	69
3^c^				72	63
4				100	94
5				96	89
6				91	84
7^c^				83	42 38
8^c^				76	63
9^c^				74	74

Reaction conditions: Substrate (1 mmol), H_2_O_2_ (3 mmol), H_2_O (1.5 mL), A-CQDs/W (1 mol %), 3 h, 90 °C.

^b^Conversions were calculated based on initial mmol of substrate.

^c^Isolated Yields.

^d^In the presence of 0.25 mmol CH_3_COOH.

The cinnamic acid’s family was tested to examine the catalytic system efficiency on cleavage electron-deficient substrate. Although extremely great attention has been paid to these substrates' oxidative cleavage, most of them have been suffering from a lack of high selectivity to produce aldehydes^47^. Fortunately, with A-CQDs/W as a catalyst, cinnamic acid (1d), 4-chlorocinnamic acid (1e) and 3-chlorocinnamic acid (1f) converted to corresponding benzaldehyde with excellent to good yield (entry 4–6).

Chalcone, as a common scaffold with a privileged structure, has been widely utilized as an efficient template for drug discovery in medicinal chemistry^48^. Therefore, the oxidative behavior of such an important alkene is worth mentioning. Luckily the double bond of *trans*-chalcone (1 g) also undergoes oxidative C = C bond cleavage to form benzaldehyde and phenylglyoxal (2 g) with 42% and 38% yield, respectively (entry 7). Finally, the styrene (1 h) and *trans*-stilbene (1i) as unfunctionalized olefins were promisingly oxidized to give approximately high yields of the benzaldehyde (entries 8, 9).

### Suggested reaction mechanism

We designed some experiments to clarify the mechanism of the reaction. First, we investigated A-CQD/W as a potent catalyst to promote the scission of C=C double bonds from a radical or nonradical pathway. Accordingly, the oxidative cleavage of C=C bonds of alkenes was performed in EtOH solvent due to it reacts quickly with the hydroxyl radical species produced during activation of oxidant. Assuming, that the reaction mechanism proceeds through the radical pathway, the cleavage reaction of alkenes would be extremely diminished or shutted down in EtOH solvent^17^. In this regard, coumarin-3-carboxylic acid was degraded to salicylaldehyde with 61% yield in EtOH under optimized condition (Table 1, entry 16). Based on these results, probably PMC proceed with the oxidative reaction from a nonradical pathway.

A review article reported by Gebbink concludes that tungsten-based catalysts cleavage alkenes to aldehydes through a kind of intermediates such as epoxides (**I**) and diols (**II**). It seems that in situ generated tungsten-bisperoxo species from WO_4_^2−^ and H_2_O_2_, are responsible for the epoxidation, ketonization, and cleavage reactions^49^.

**Scheme 3:**
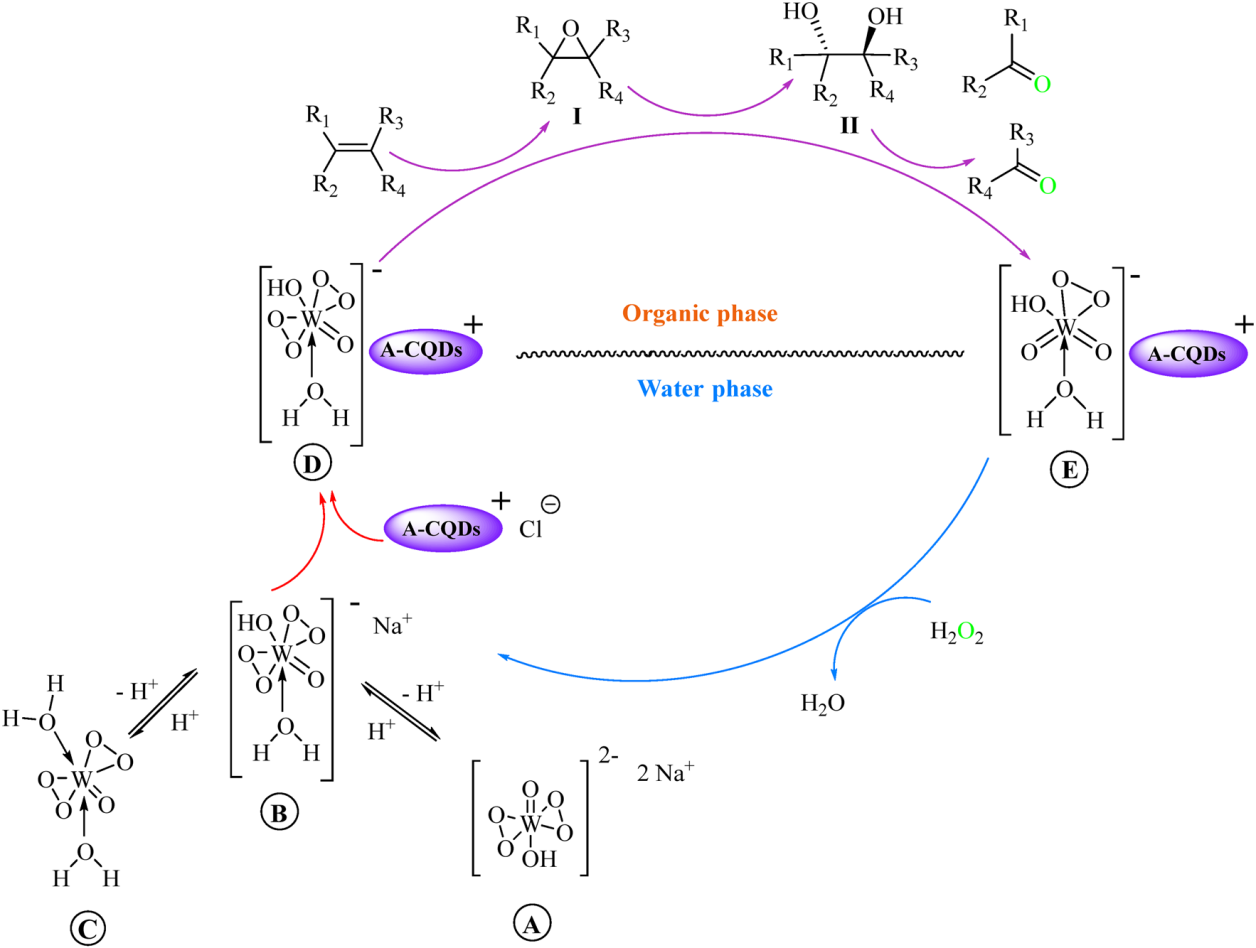
Reaction pathway for the oxidative cleavage reactions of alkenes in the presence of the A-CQDs/W and H_2_O_2_.

In this regard, we proposed a mechanism for oxidative cleavage of C=C bonds of alkenes to aldehydes in the presence of the A-CQDs/W and H_2_O_2_ (Scheme 3). Interestingly, the carboxylic acid functional groups anchored on A-CQDs were found to be beneficial to create an appropriate acidity environment, which are necessary to activate bisperoxotungstate compound (**A**) to mono- and di-protonated form **B** and **C,** respectively. The neutral bisperoxo compound **C** as a minor component in media, was feeble to transfer into the organic phase. However, mono anionic compound **B** is a more active species and capable of interacting with A-CQDs to form complex **D**. So, A-CQD acts as an aqueous–organic PTC of the active species **B**^49^. Hence, in the organic layer, the reactive complex **D** participates in oxidative cleavage reaction to form aldehyde and **E**. The monoperoxo tungstate ion **E** is re-oxidized by H_2_O_2_ to continue the oxidation cycle.

Based on this mechanism, the catalytic activity might be highly influenced by the reaction medium’s acidity. To prove the effect of acidic groups on oxidation efficiency, the oxidative cleavage reaction of *trans*-stilbene as a nonacidic substrate and coumarin-3-carboxylic acid as an acidic one, was examined in the presence and absence of different acidic additives under optimized condition (Table 4).

**Table 4:** Investigation of oxidative cleavage of *trans*-stilbene and coumarin-3-carboxylic acid under optimum conditions in the presence of additives.

Entry	Substrate	Additive (mmol)	Conversion (%)^a^	Yield (%)^b^
1	*trans*-stilbene	–	63	58
2	*trans*-stilbene	ClCH_2_COOH (0.25)	79	74
3	*trans*-stilbene	ClCH_2_COOH (0.5)	56	49
4	Coumarin-3-carboxylic acid	-	95	86
5	Coumarin-3-carboxylic acid	ClCH_2_COOH (0.25)	73	65
6	Coumarin-3-carboxylic acid	H_3_PO_4_ (0.15)	50	31
7	Coumarin-3-carboxylic acid	CH_3_COOH (0.2)	45	33
8	Coumarin-3-carboxylic acid	HCl (0.1)	30	21
9	Coumarin-3-carboxylic acid	H_2_SO_4_ (0.1)	28	23
10	Coumarin-3-carboxylic acid	NaHCO_3_ (0.17)	Trace	Trace

Reaction conditions: Coumarin-3-carboxylic acid/ *trans*-stilbene (1 mmol), H_2_O_2_ (3 mmol), H_2_O (1.5 mL), A-CQDs/W (1 mol %), 3 h, 90 °C.

^a^Conversions were calculated based on initial mmol of substrate.

^b^Isolated Yields.

*trans*-stilbene undergoes oxidative reaction in the presence of A-CQDs/W to generate benzaldehyde product with 58% yield (Table 4, entry 1). The best result of *trans*-stilbene oxidative cleavage is observed when 0.25 mmol chloroacetic acid was used as an acidic substrate (entry 2). However, increasing the amount of additive to 0.5 mmol reduces the yield to 49% (entry 3).

Expectedly, in the case of coumarin-3-carboxylic acid, as an acidic substrate, the presence of acidic additives resulted in decreasing both conversion and yield (entries 4–10). Probably, under these much acidic reaction conditions, the di-protonated form of bisperoxotungstate species (**C)** is presented as the major tungstate components. Although these components are reactive in an aqueous media, they cannot transfer into the organic layer under the biphasic systems (Scheme 3)^49^. Besides, replacing acidic additives with alkaline ones leads to nothing (entry 10). It can be explained probably by unproductive decomposition of H_2_O_2_ in basic conditions^50^ or by the presence of de-protonated bisperoxotungstate compound (**A**) as the dominant form of tungstate with poor catalytic activity even in the presence of A-CQDs. Therefore, the nature of the oxidative cleavage reaction in the case of non-acidic substrate is not similar to acidic substrates. The results are in good agreement with results reported by Noyori’s group^49^.

**Figure 3:**
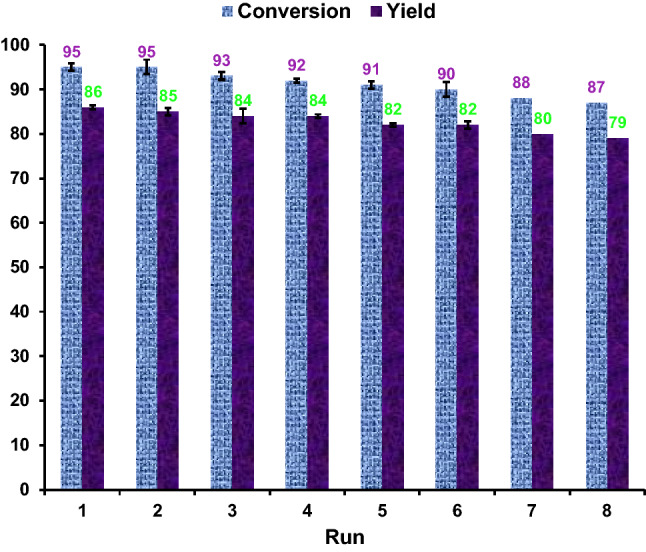
Recycling of the A-CQDs/W catalyst in oxidative cleavage of coumarin-3-carboxylic acid.

The reusability is a desirable characteristic of catalysts in commercial and industrial applications to reduce environmental pollution and production price considerably. Therefore, the recovery and reusability of the A-CQDs/W catalyst were surveyed by the oxidative cleavage of coumarin-3-carboxylic acid under the optimized reaction conditions as a test model. After the reaction was completed and allowed to be cooled to room temperature, the catalyst was readily recovered from the reaction mixture by solvent extraction with ethyl acetate. The aqueous layer was separated and dried under vacuum overnight to obtain the A-CQDs/W, and the next catalytic run was carried out using this recovered catalyst. Eight consecutive recycling experiments clearly demonstrated that A-CQDs/W displayed excellent stability and reusability by producing aldehyde at high yield even after 8 runs (first run: 86% yield, eighth run: 79% yield, Fig. 3). Further evidence in support of the stability of the A-CQDs/W was achieved from ICP analysis. In this regard, the leaching of W during the catalytic reactions was determined using ICP analysis. The ICP analysis indicated that only about 2 wt % of W content leached after 8 runs (the fresh catalyst and the recovered catalyst after 8 runs contain 1.06 mmol g^−1^ and 1.02 mmol g^−1^ of W, respectively); which clearly demonstrates the stability of the A-CQDs/W.

## Conclusion

We have presented conclusive evidence that A-CQD/W is a powerful PMC for selective oxidative scission of C=C into aldehydes without overoxidation to acid in the presence of H_2_O_2_ in water as a green solvent. With the intrinsic ability based on functionalized graphene structure, sub-nanometric sizes, amphiphilic architecture, the catalyst promises accessibility in polar-nonpolar reaction media with high mass transferability to the biphasic system without additives and PTC. In addition, it can be resumed from the reaction media by simple extraction or membrane. On the other hand, the A-CQDs act as a vehicle for pick up catalytically active tungsten species, to transfer between aqueous and organic phases. Fortunately, the A-CQDs enhanced the tungsten species' stability and solubility in the reaction medium without discernible loss of catalytic efficiency even after eight catalytic reaction cycles. The catalyst was characterized by complementary techniques including FT-IR, UV–vis, ^1^H NMR, ^13^C NMR, FE-SEM, TEM, HR-TEM, XRD, EDX, fluorescence spectroscopy, XPS, TGA, and ICP analysis. Importantly, we have proven that the cleavage reaction progress through the nonradical pathway, and the dominant intermediate for oxidation is mono anionic compound **B**. Finally, compared to the previous oxidative scission of olefin systems, this procedure has the advantages of having a green oxidant, clean, safe, low cost, excellent selectivity for aldehydes without overoxidation, high yields and reusability of the catalyst. We forcefully recommended that the current practices using harsh and toxic procedures should be changed by these green and straightforward catalytic processes. All in All, this PMC will be widely expected to open new windows for utilizing various kinds of future catalytic reactions.

## Supplementary Information

Supplementary Information.
