# Supplementary material for: Pseudohomogeneous metallic catalyst based on tungstate-decorated amphiphilic carbon quantum dots for selective oxidative scission of alkenes to aldehyde
Source: Sci Rep. 2021 Feb 24;11:4411. doi: 10.1038/s41598-021-83863-0 (PMC7904908; doi:10.1038/s41598-021-83863-0)
Supplement: Supplementary file 1 — Supplementary Information. [file 41598_2021_83863_MOESM1_ESM.docx]

*Supporting Information*

**Pseudohomogeneous Metallic Catalyst Based on Tungstate-Decorated Amphiphilic Carbon Quantum Dots for Selective Oxidative Scission of Alkenes to Aldehyde**

Aram Rezaei,* ^§ɸ^ Leila Hadian-Dehkordi, ^§ɸ^ Hadi Samadian,^§^ Mehdi Jaymand,^§^ Homa Targhan,^£^ Ali Ramazani,^∆^ Hadi Adibi,^ƛ^ Xiaolei Deng, ^¥,€^ Lingxia Zheng^¥,€^ and Huajun Zheng^¥,€^

§ Nano Drug Delivery Research Center, Health Technology Institute, Kermanshah University of Medical Sciences, Kermanshah, Iran. E-mail: aram.rezaei@gmail.com, [aram.rezaei@kums.ac.ir](mailto:aram.rezaei@kums.ac.ir)

£ Department of Organic Chemistry, Faculty of Chemistry, University of Razi, Kermanshah 67149-67346, Iran.

∆ Department of Chemistry, University of Zanjan, Zanjan, Iran.

ƛ Pharmaceutical Sciences Research Center, Health Institute, Kermanshah University of Medical Sciences, Kermanshah, Iran.

¥ Department of Applied Chemistry, Zhejiang University of Technology, Hangzhou 310032, China.

€ State Key Laboratory Breeding Base of Green Chemistry Synthesis Technology, Zhejiang University of Technology, Hangzhou 310032, China.

ɸ These authors contributed equally to this work as first authors.

Table of Contents

| List of contents | Page |
| --- | --- |
| Title, author’s name and address | S1 |
| Result and discussion:  Figure S1. FT-IR spectra of the A-CQDs and A-CQDs/W | S3 |
| Result and discussion:  Figure S2. TGA thermograms of A-CQDs and A-CQDs/W | S4 |
| Result and discussion:  Figure S3. ^1^H NMR and ^13^C NMR images of the A-CQDs | S5 |
| Result and discussion:  Figure S4. FE-SEM image of the A-CQDs/W | S6 |
| Result and discussion:  Figure S5. Elemental mappings of A-CQDs/W; and EDX patterns of A-CQDs/W. | S7 |
| Result and discussion:  Figure S6. The emission spectra of A-CQDs/W and UV-vis for A-CQDs and A-CQDs/W | S8 |
| Result and discussion:  Figure S7. XRD pattern of the A-CQDs/W | S9 |
| Result and discussion:  Figure S8. Photographs of emission of the A-CQDs/W | S10 |


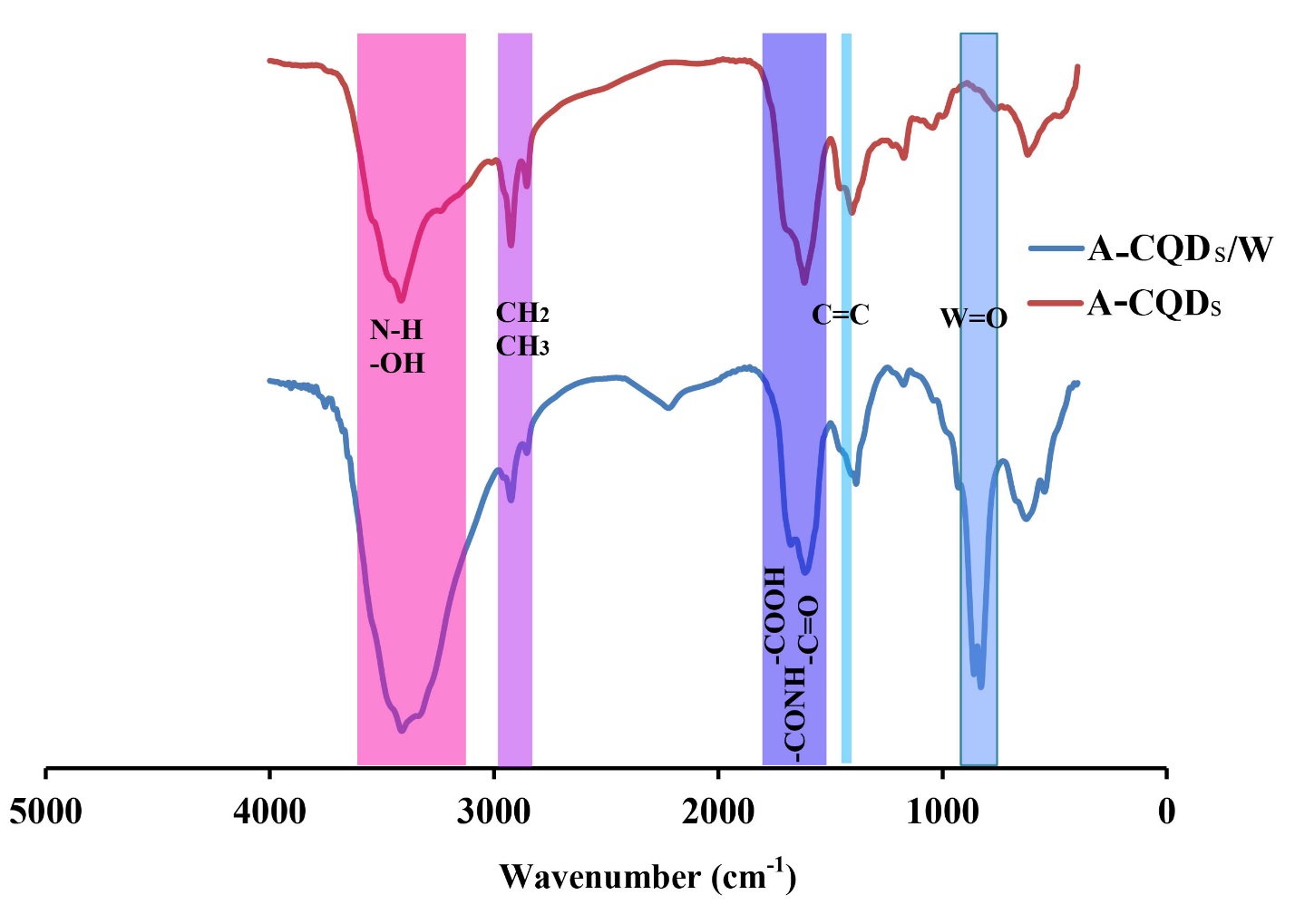


**Figure S1.** FT-IR spectra of the A-CQDs and A-CQDs/W,


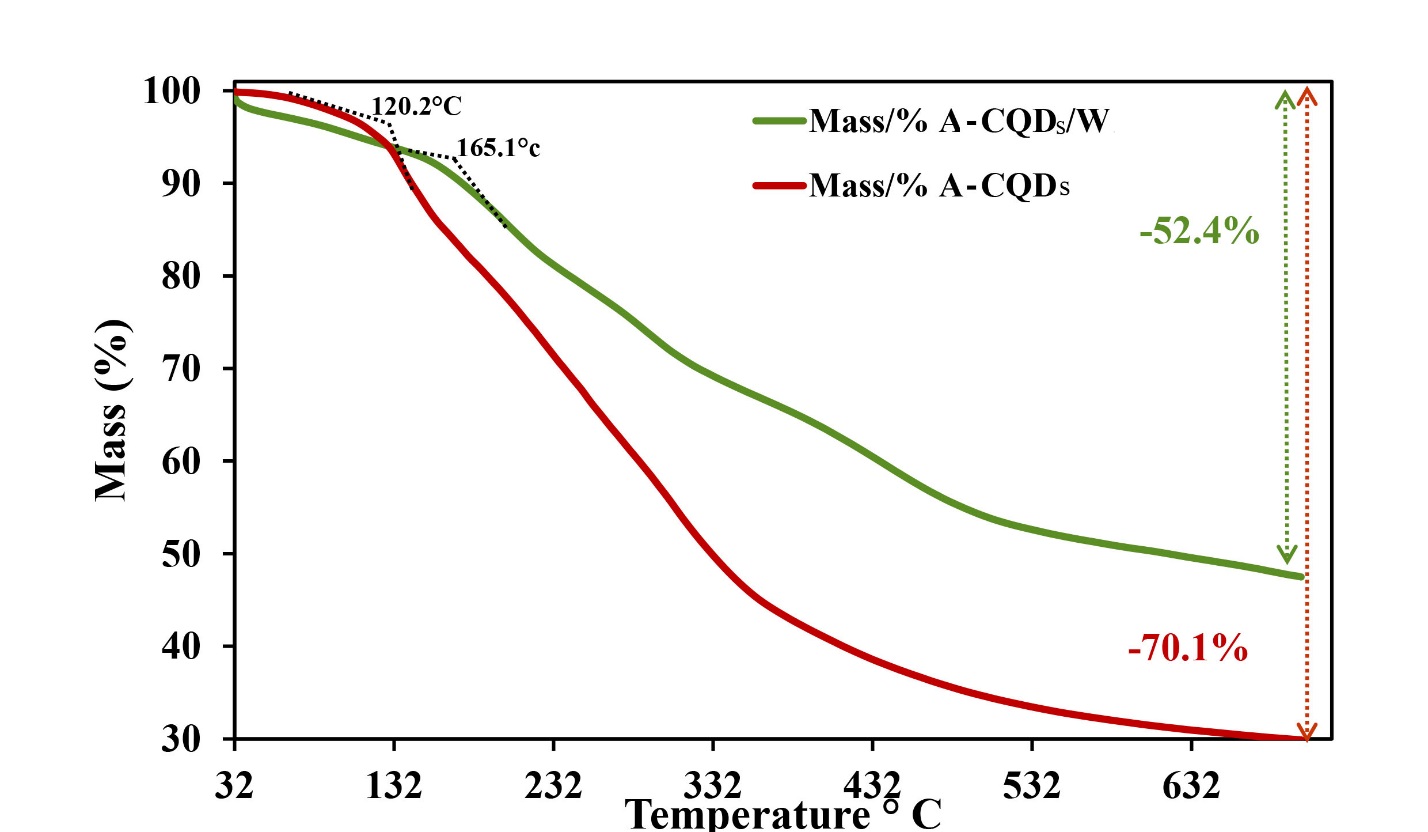


**Figure S2.** TGA thermograms of A-CQDs and A-CQDs/W.


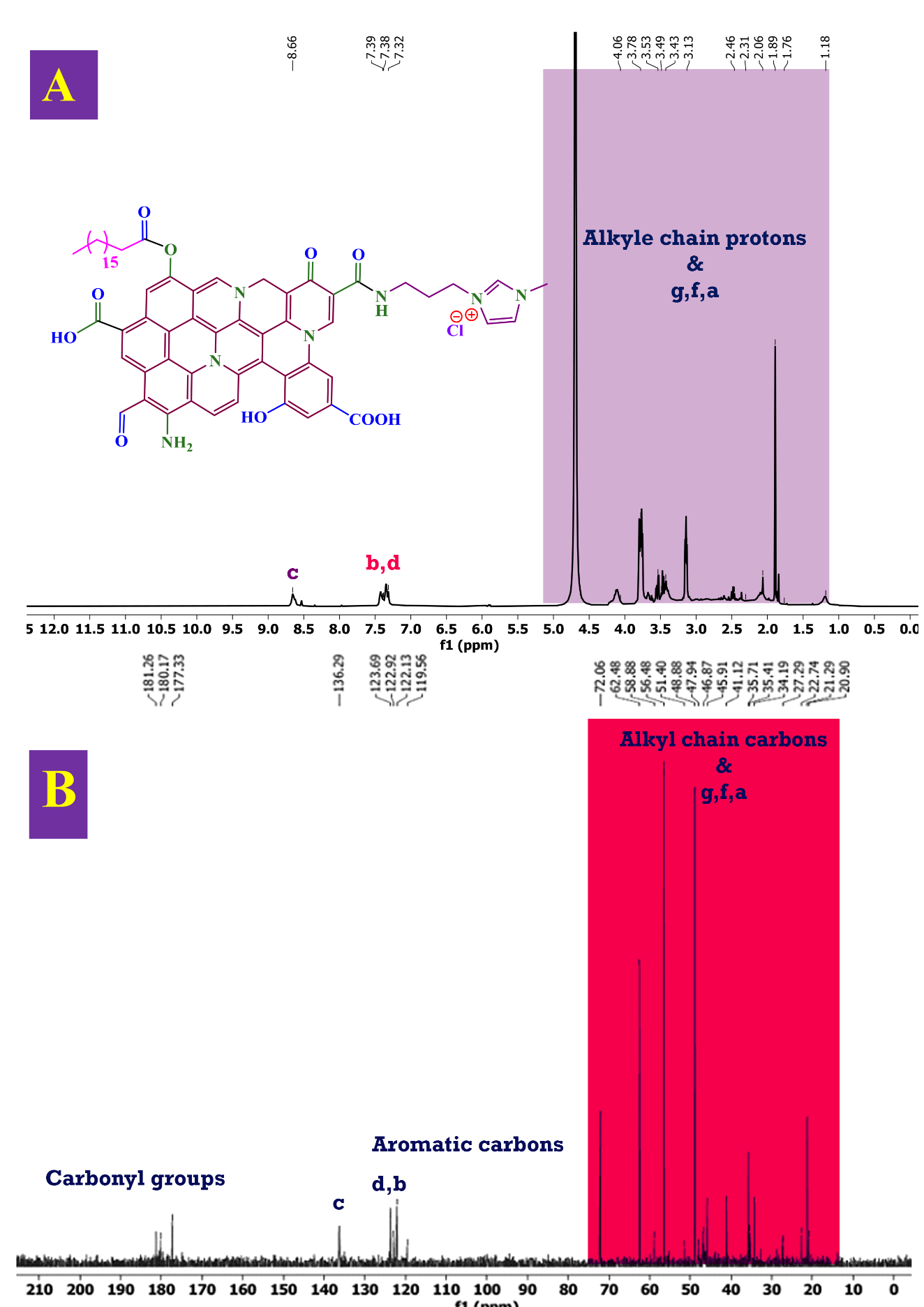


**Figure S3.** **(A)** ^1^H NMR and **(B)** ^13^C NMR images of the A-CQDs


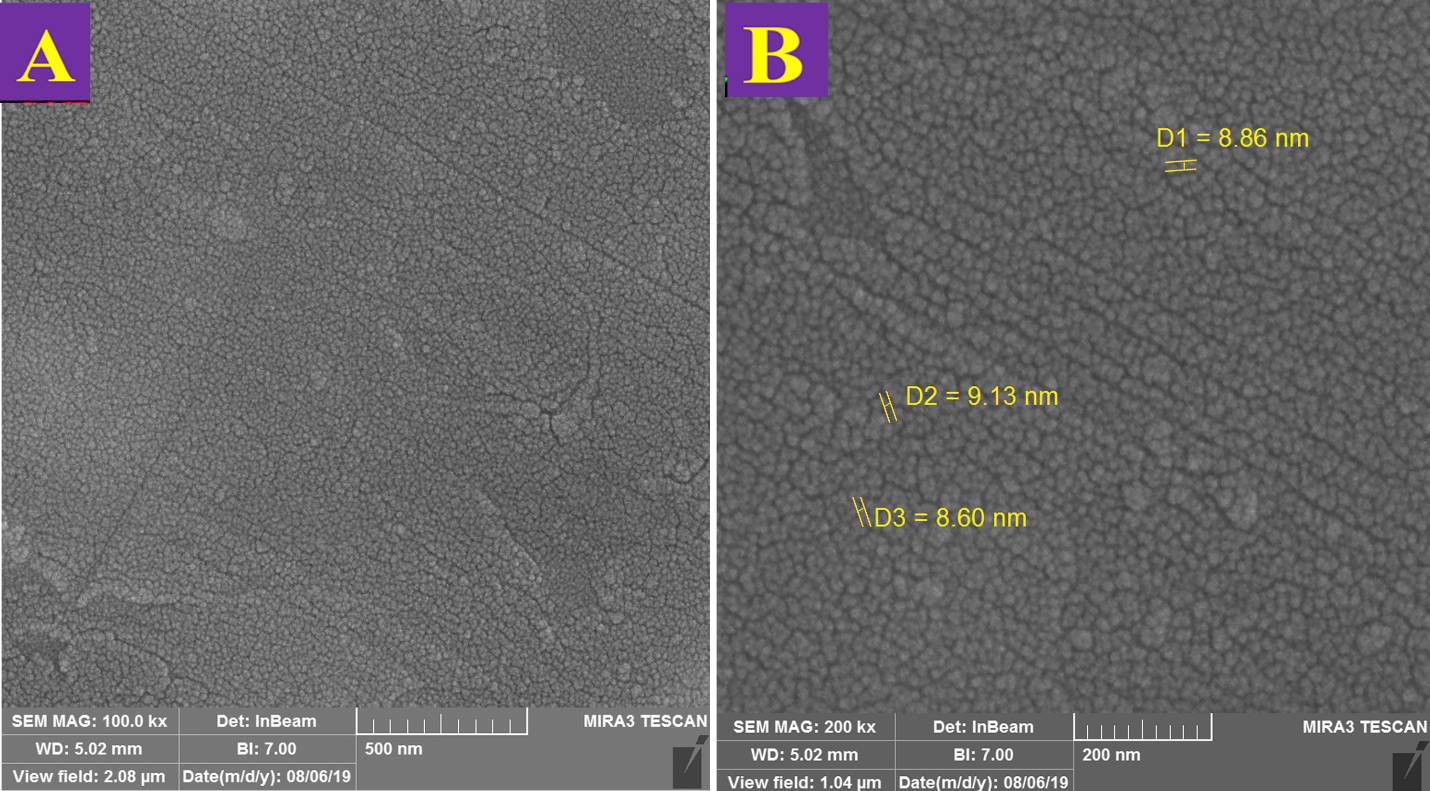


**Figure S4.** FE-SEM image of the A-CQDs/W

**
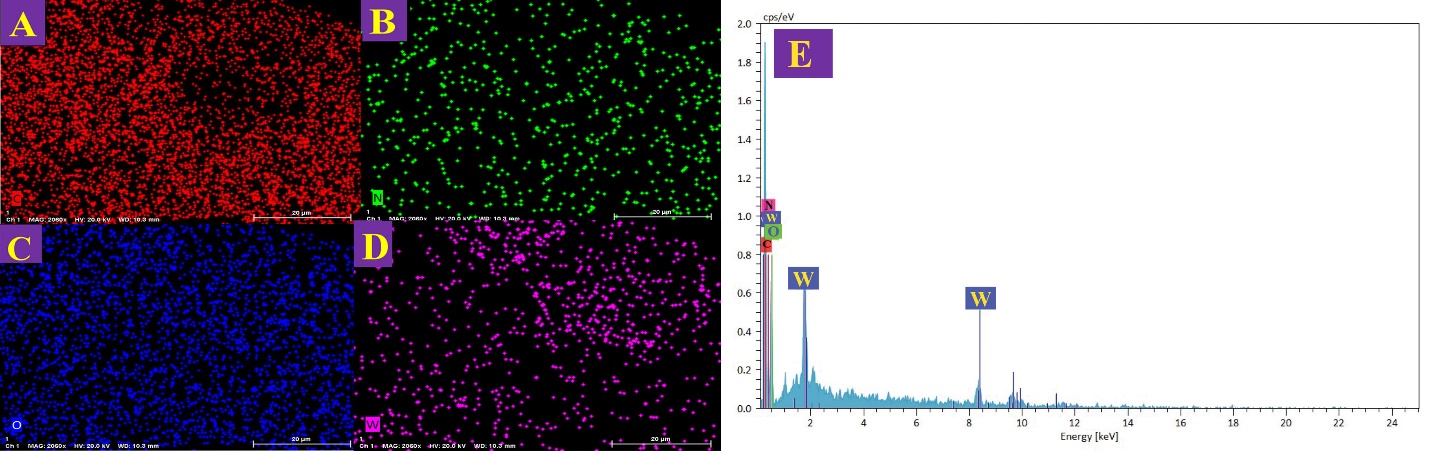
**

**Figure S5.** (**A-D**) Elemental mappings of A-CQDs/W; and **(E)** EDX patterns of A-CQDs/W.


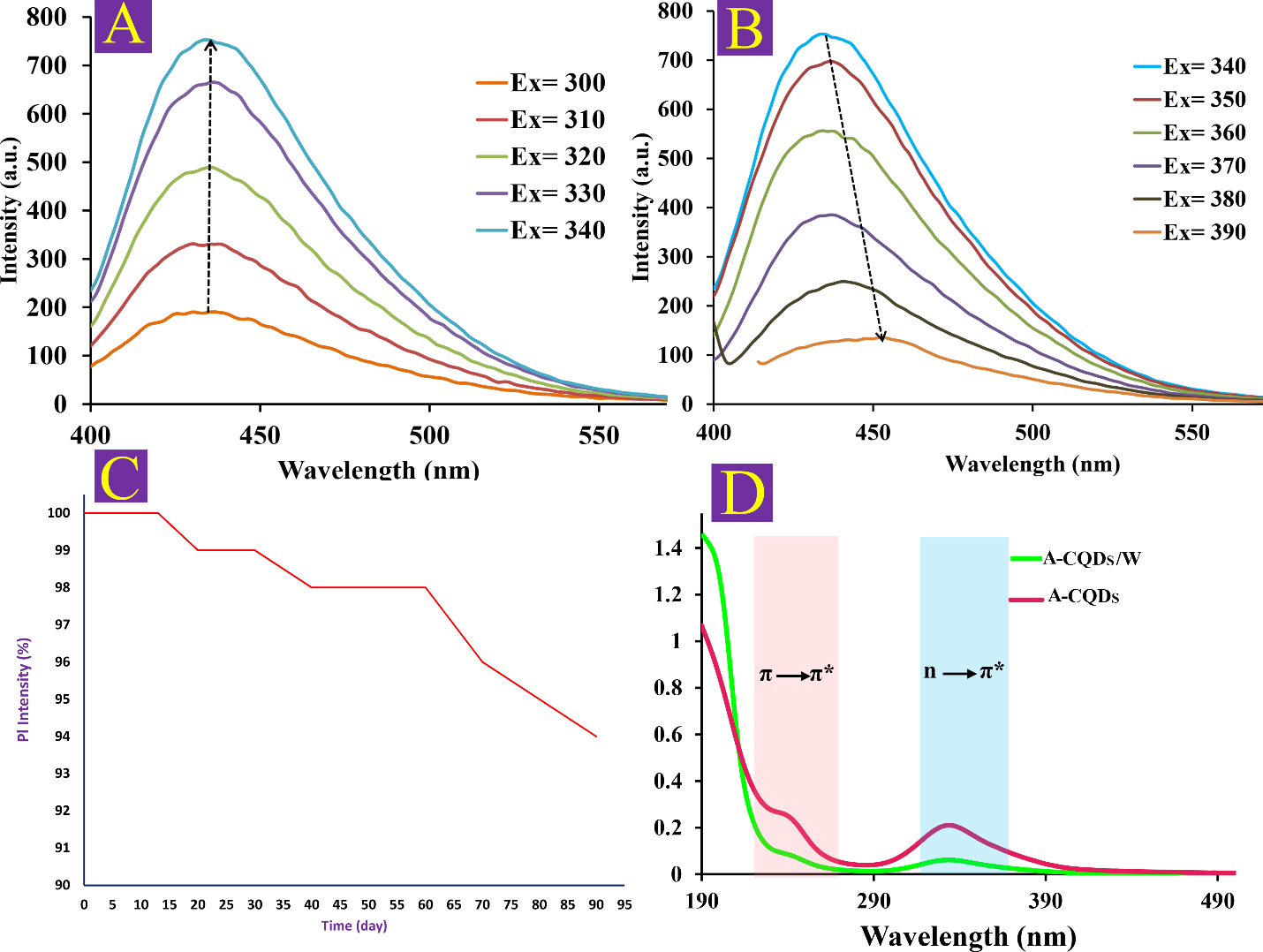


**Figure S6.** **(A)** The emission spectra of A-CQDs/W with increasing excitation wavelengths from 300 nm to 340 nm in 10 nm increments, **(B)** The emission spectra of A-CQDs/W with increasing excitation wavelengths from 340 nm to 390 nm in 10 nm increments, **(C)** Fluorescence stability of A-CQDs/W vs time, and **(D)** UV-vis absorbance spectra of A-CQDs and A-CQDs/W.


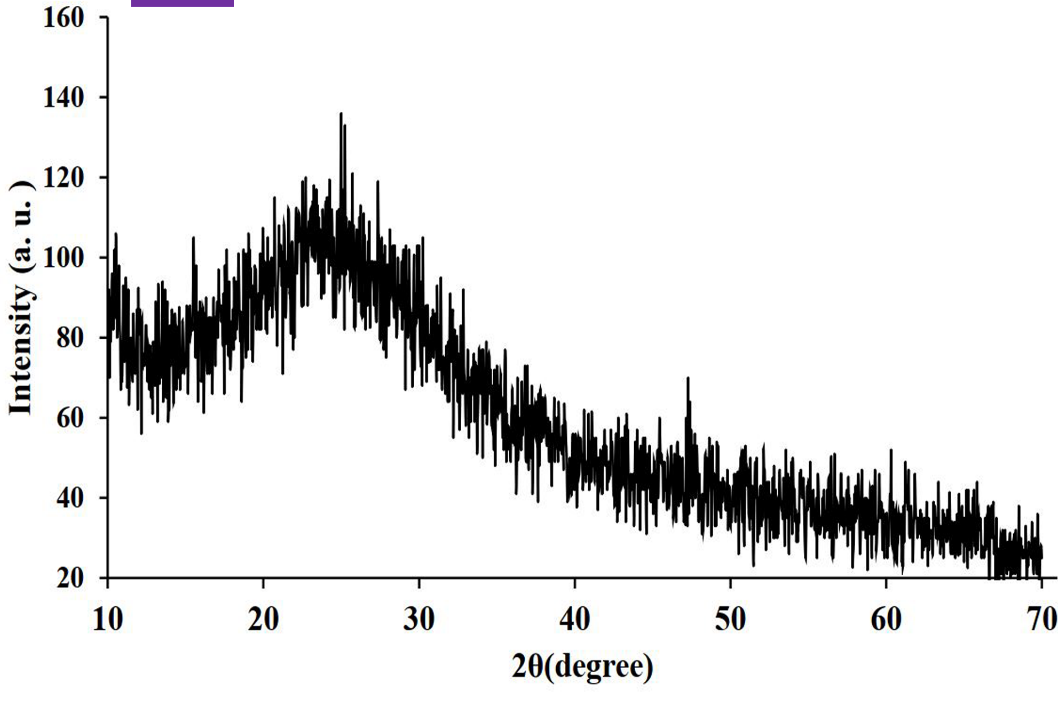


**Figure S7.** XRD pattern of the A-CQDs/W

**
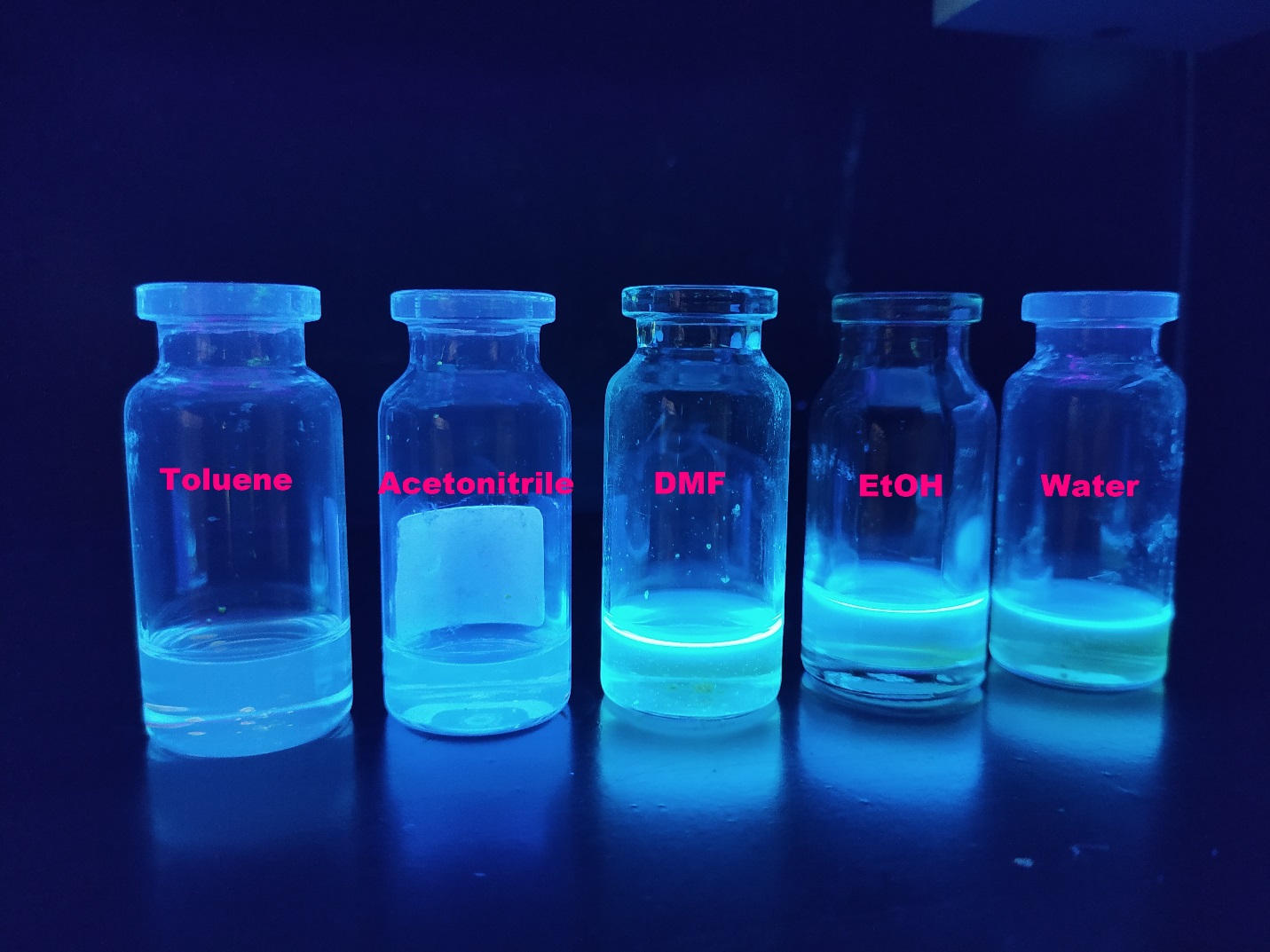
**

**Figure S8**. Photographs of emission of the A-CQDs/W dissolved in a range of common laboratory solvents under 365 nm UV irradiation.
